# Supplementary material for: Global Trends in Phytohormone Research: Google Trends Analysis Revealed African Countries Have Higher Demand for Phytohormone Information
Source: Plants (Basel). 2020 Sep 22;9(9):1248. doi: 10.3390/plants9091248 (PMC7570059; doi:10.3390/plants9091248)
Supplement: Supplementary file 1 [file plants-09-01248-s001.zip › Supplementary Materials/Supplementary Figure 3.pptx]

## Slide 1
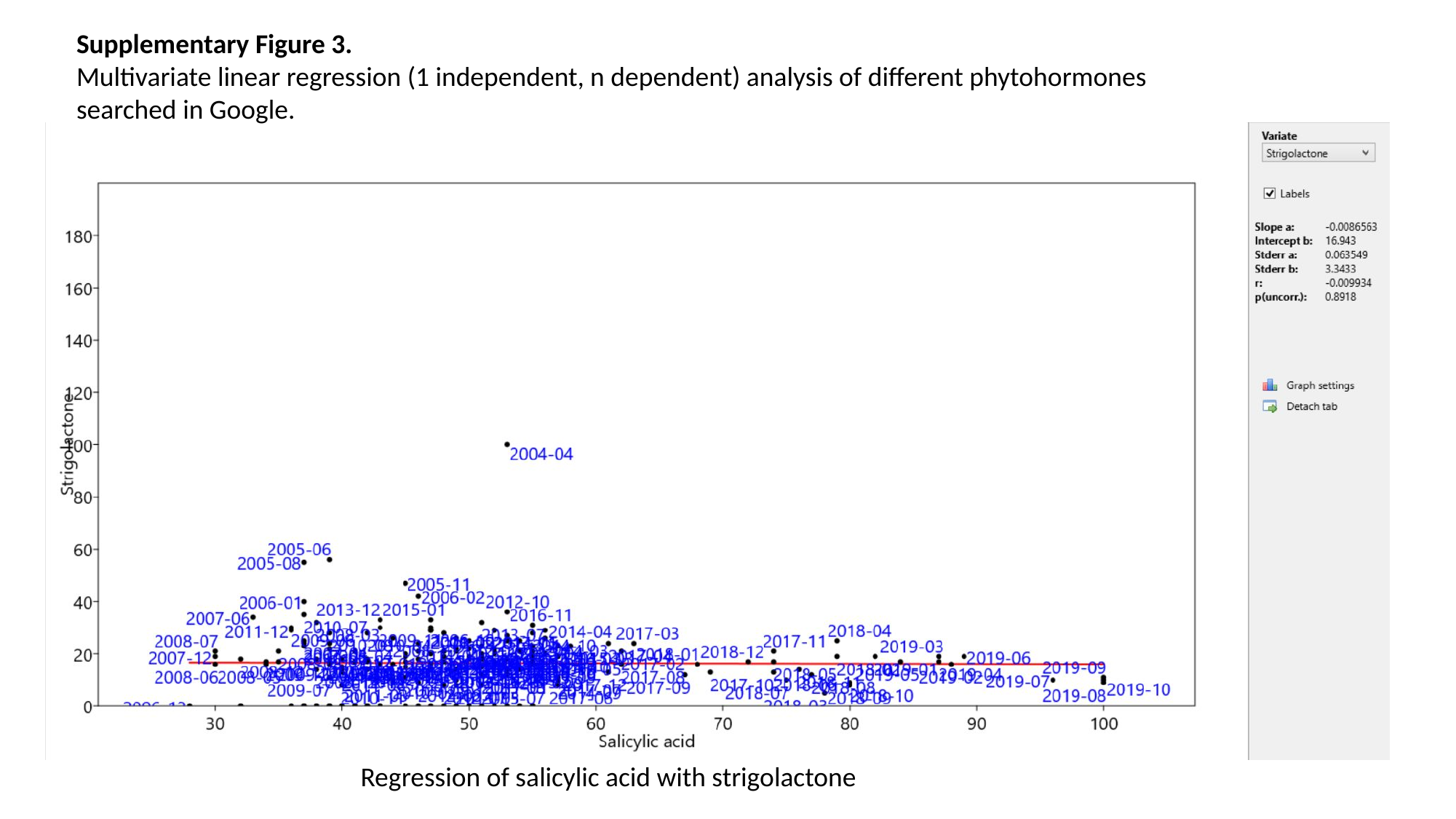

Supplementary Figure 3.
Multivariate linear regression (1 independent, n dependent) analysis of different phytohormones searched in Google.
Regression of salicylic acid with strigolactone

## Slide 2
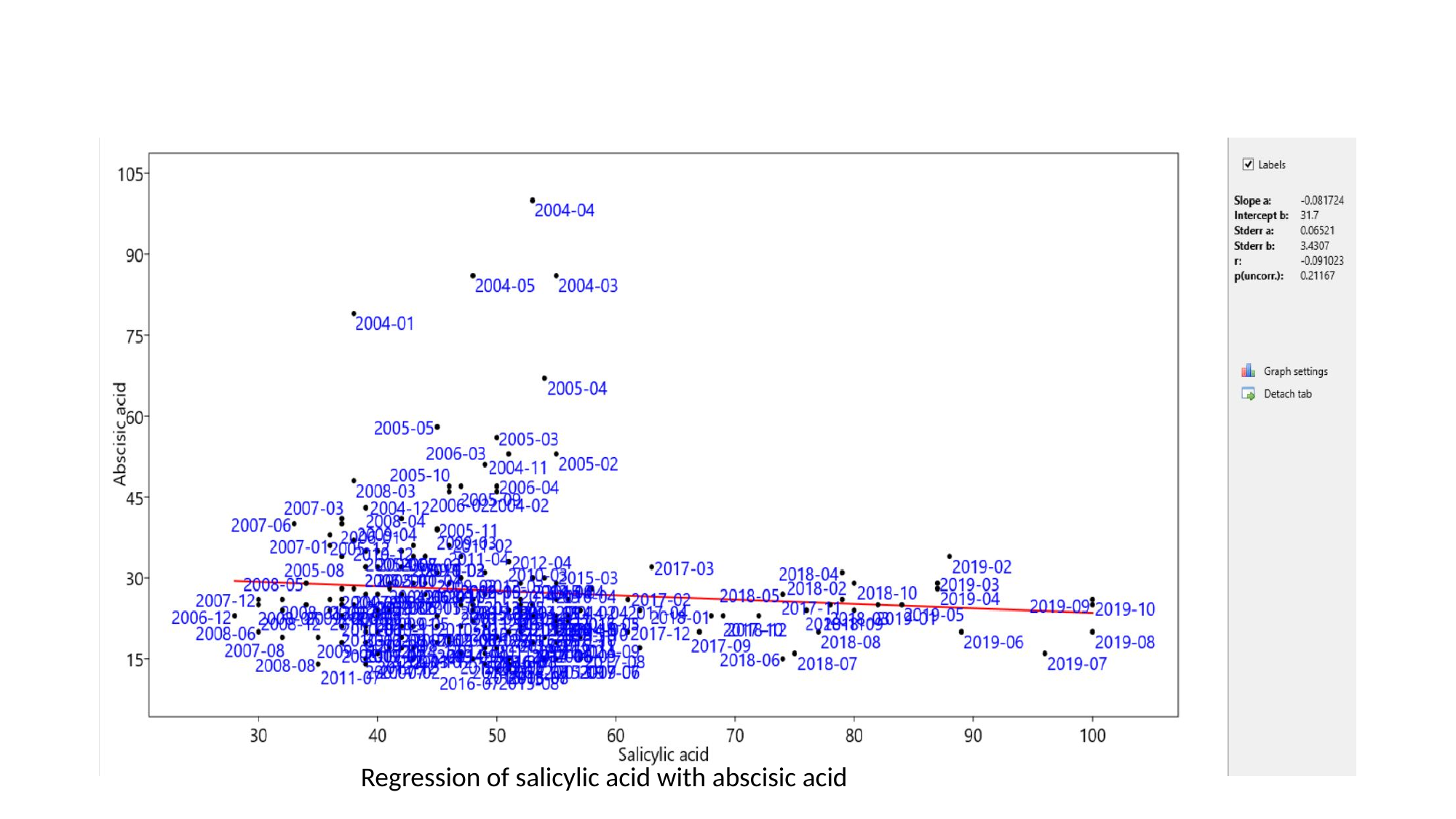

Regression of salicylic acid with abscisic acid

## Slide 3
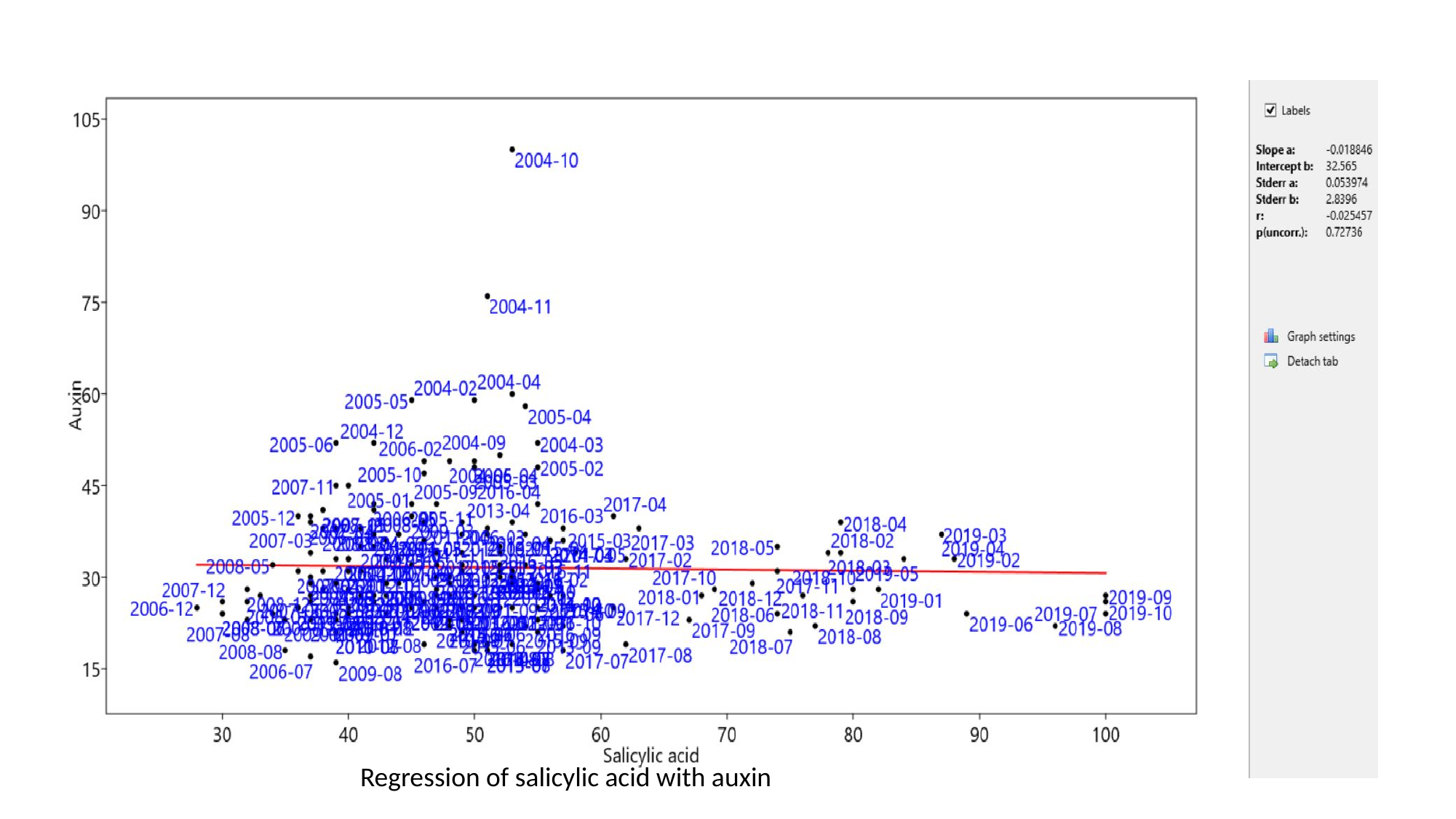

Regression of salicylic acid with auxin

## Slide 4
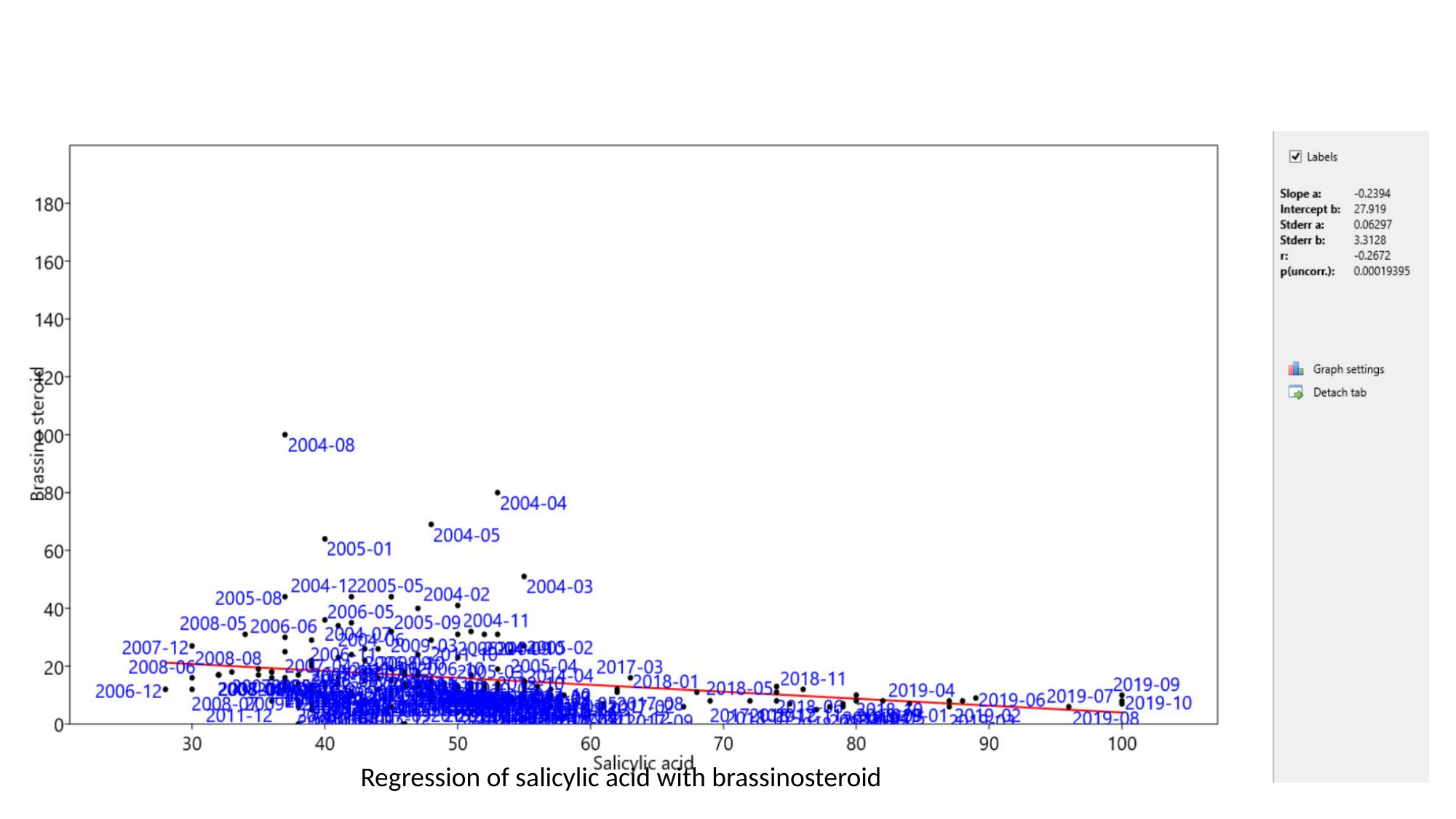

Regression of salicylic acid with brassinosteroid

## Slide 5
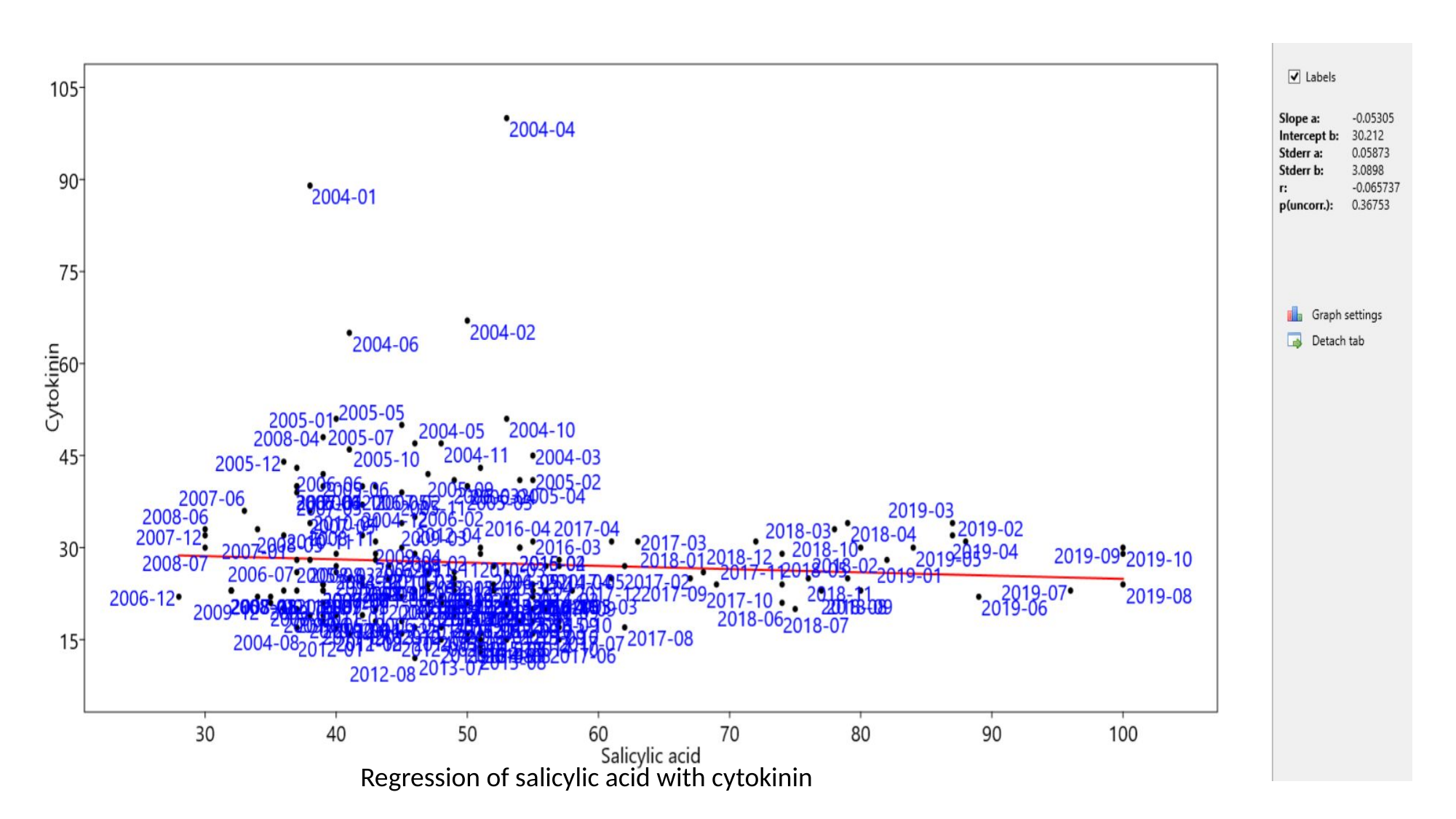

Regression of salicylic acid with cytokinin

## Slide 6
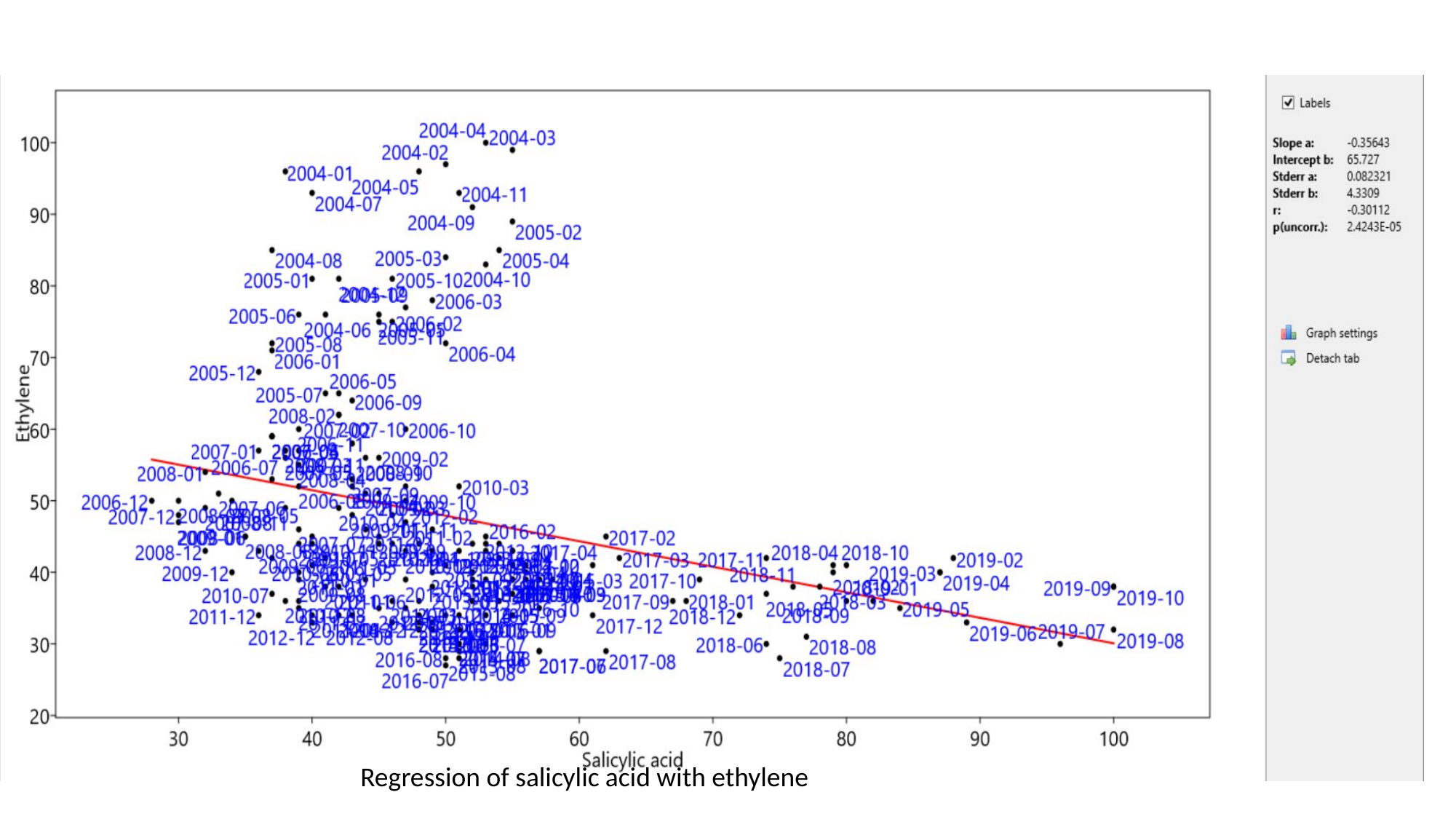

Regression of salicylic acid with ethylene

## Slide 7
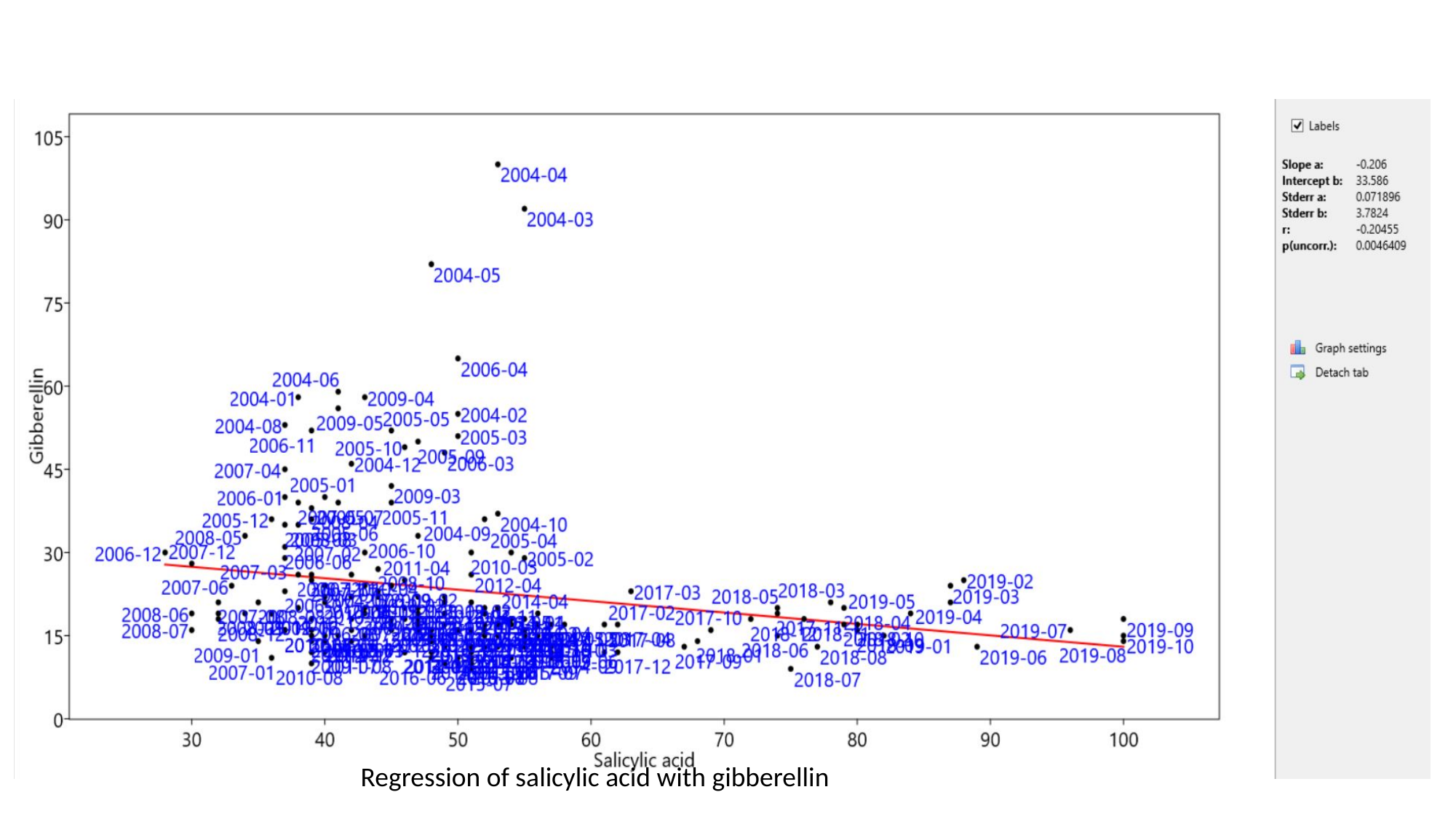

Regression of salicylic acid with gibberellin

## Slide 8
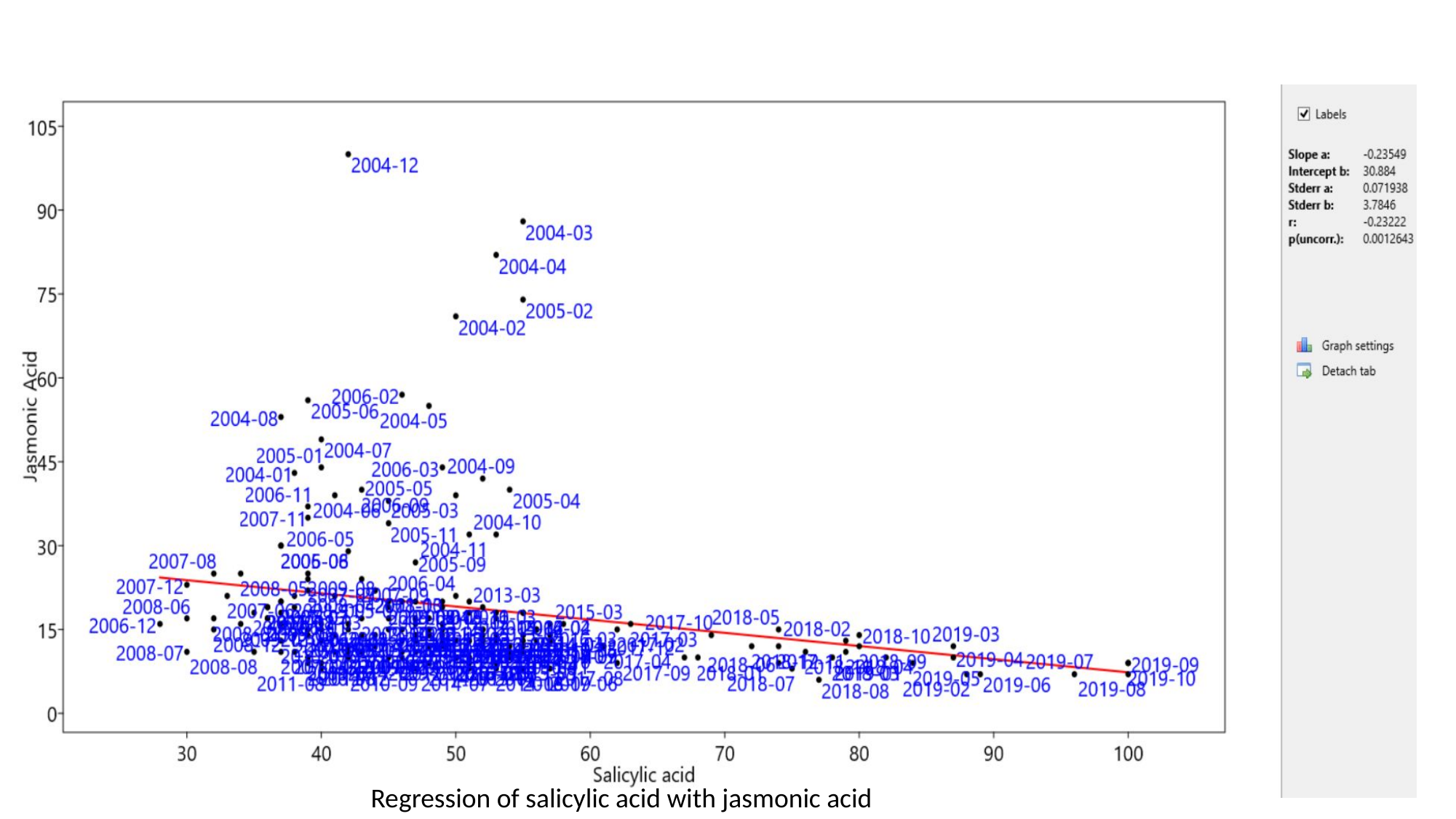

Regression of salicylic acid with jasmonic acid
