# Supplementary figures and images for: Global Trends in Phytohormone Research: Google Trends Analysis Revealed African Countries Have Higher Demand for Phytohormone Information
Source: Plants (Basel). 2020 Sep 22;9(9):1248. doi: 10.3390/plants9091248 (PMC7570059; doi:10.3390/plants9091248)

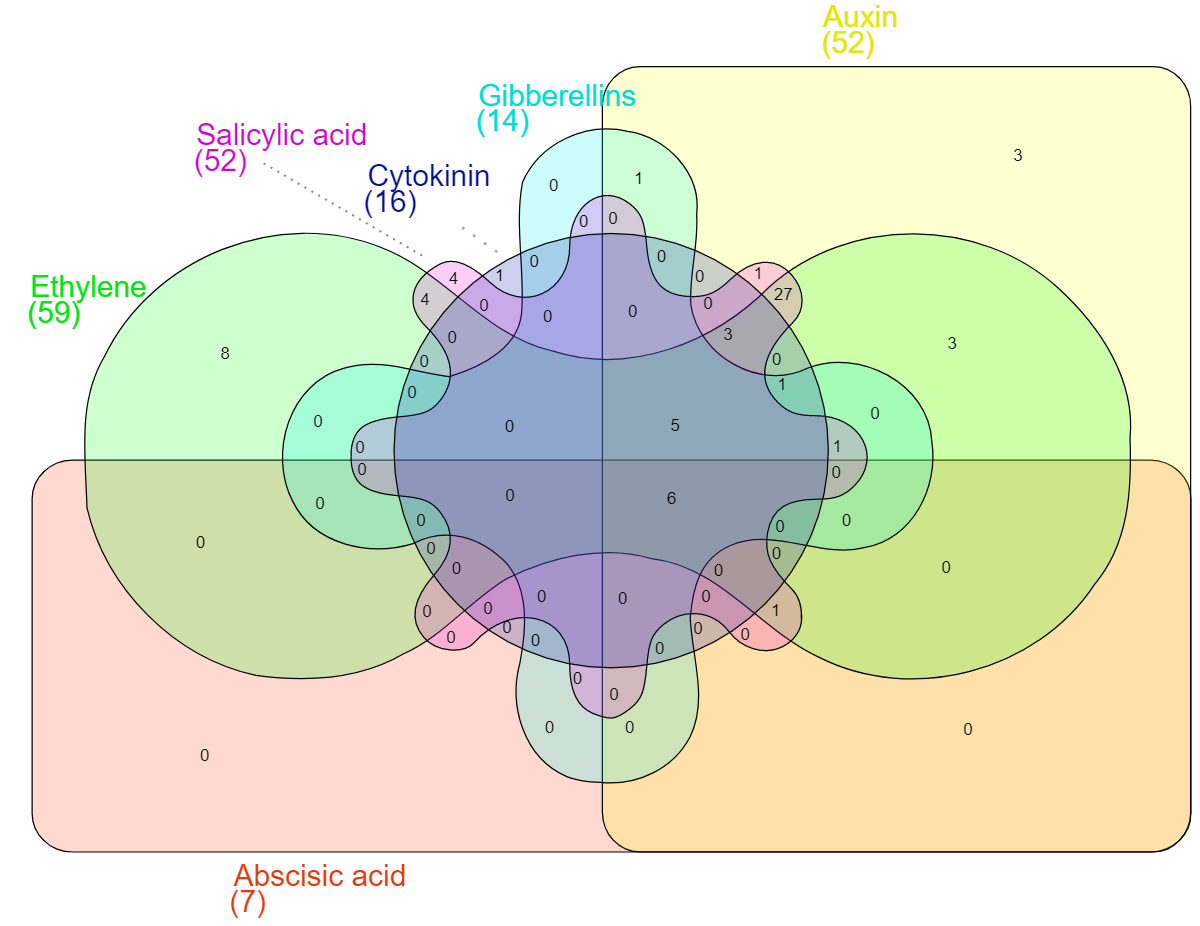

Supplement: Supplementary file 1 [file plants-09-01248-s001.zip › Supplementary Materials/Supplementary Figure 2.png]
